# Analysis of 17,576 Potentially Functional SNPs in Three Case–Control Studies of Myocardial Infarction

**DOI:** 10.1371/journal.pone.0002895

**Published:** 2008-08-06

**Authors:** Dov Shiffman, John P. Kane, Judy Z. Louie, Andre R. Arellano, David A. Ross, Joseph J. Catanese, Mary J. Malloy, Stephen G. Ellis, James J. Devlin

**Affiliations:** 1 Celera, Alameda, California, United States of America; 2 Cardiovascular Research Institute, University of California San Francisco, San Francisco, California, United States of America; 3 Department of Cardiovascular Medicine, The Cleveland Clinic Foundation, Cleveland, Ohio, United States of America; Innsbruck Medical University, Austria

## Abstract

Myocardial infarction (MI) is a common complex disease with a genetic component. While several single nucleotide polymorphisms (SNPs) have been reported to be associated with risk of MI, they do not fully explain the observed genetic component of MI. We have been investigating the association between MI and SNPs that are located in genes and have the potential to affect gene function or expression. We have previously published studies that tested about 12,000 SNPs for association with risk of MI, early-onset MI, or coronary stenosis. In the current study we tested 17,576 SNPs that could affect gene function or expression. In order to use genotyping resources efficiently, we staged the testing of these SNPs in three case–control studies of MI. In the first study (762 cases, 857 controls) we tested 17,576 SNPs and found 1,949 SNPs that were associated with MI (P<0.05). We tested these 1,949 SNPs in a second study (579 cases and 1159 controls) and found that 24 SNPs were associated with MI (1-sided P<0.05) and had the same risk alleles in the first and second study. Finally, we tested these 24 SNPs in a third study (475 cases and 619 controls) and found that 5 SNPs in 4 genes (*ENO1, FXN* (2 SNPs), *HLA-DPB2,* and *LPA*) were associated with MI in the third study (1-sided P<0.05), and had the same risk alleles in all three studies. The false discovery rate for this group of 5 SNPs was 0.23. Thus, we have identified 5 SNPs that merit further examination for their potential association with MI. One of these SNPs (in *LPA*), has been previously shown to be associated with risk of cardiovascular disease in other studies.

## Introduction

Myocardial infarction (MI) is a prevalent and often fatal consequence of coronary heart disease. Each year approximately 865 thousand Americans are diagnosed with MI and about 180 thousand die from the disease [1]. MI occurs when thrombosis—precipitated by ruptured or eroded atherosclerotic plaque—leads to acute ischemia and subsequent necrosis of the myocardium.

Risk factors for MI include age, sex, elevated LDL-cholesterol, hypertension, low HDL-cholesterol, smoking, type 2 diabetes, and family history of cardiovascular disease. Risk of MI has a genetic component as evidenced by large twin studies [2] which showed that death from cardiovascular disease is more highly correlated among identical twins than fraternal twins. Among genetic variants that are associated with MI, some can affect traditional risk factors [3], [4] but for others the underlying biological explanation for the association is not known [5], [6].

The identification of genetic variants that are associated with MI is a challenging task, because variants that are associated with MI are expected to only modestly increase the risk of MI, and because a large number of variants could potentially be tested. Thus, very large studies are needed to detect modest association and account for multiple testing confidently [7]. We have limited the magnitude of multiple testing in the past [8]–[10] by testing SNPs with high prior probability for association with MI. Previously, we described the results from investigating ∼12,000 such SNPs [8]–[10]. Here we asked if we could identify SNPs that are associated with MI by investigating ∼17,000 SNPs. We identified 5 SNPs that appear to be associated with MI and should be investigated in additional studies of MI.

## Methods

### Objectives

To identify genetic polymorphisms associated with MI, we interrogated three case–control studies comprising cases with a history of MI and controls without a history of MI. The first two case–control studies (Study 1 and Study 2) identified SNPs nominally associated with MI. The hypotheses that these SNPs are associated with MI were tested in Study 3. We determined the allele frequency of each SNP in pools of case and control DNA prior to determining the genotype of a smaller number of SNPs for all individual DNA samples.

### Participants

Participants in Study 1 and Study 2 were enrolled between July 1989 and May 2005 by the University of California, San Francisco (UCSF) Genomic Resource in Arteriosclerosis. UCSF samples received at the Celera genotyping facility by May 2004 were considered for inclusion in Study 1. Samples that arrived past that date were considered for Study 2. Cases in Study 1 and Study 2 included patients who had undergone diagnostic or interventional cardiac catheterization and patients of the UCSF Lipid Clinic. Controls were enrolled by the UCSF Genomic Resource in Arteriosclerosis and included UCSF staff, patients of UCSF Clinics, and senior citizens who participated in physical activities at regional community centers and events for senior citizens. A history of MI for Study 1 cases was verified by a clinical chart review or by *The International Classification of Diseases, 9^th^ Revision* (ICD9) codes 410 or 411 in the patient records. MI status for Study 2 cases was determined by ICD9 codes 410 or 411 or by a self-reported history of MI. To characterize the accuracy of these self–reported histories, medical record review for a sample of Study 2 cases resulted in verification of the self–reported MI status for 98% of the sample (verification by electrocardiogram, cardiac enzymes or imaging). Controls had no history of MI, diabetes or symptomatic vascular disease. All participants of Study 1 and Study 2 chose Caucasian as their ethnicity in response to a multiple-choice questionnaire.

Participants in Study 3 were patients of the Cleveland Clinic Foundation (CCF) Heart Center who had undergone diagnostic or interventional cardiac catheterization between July 2001 and March 2003 and enrolled in the Genebank at Cleveland Clinic Study. A history of MI was verified by electrocardiogram, cardiac enzymes, or perfusion imaging. Controls had less than 50% coronary luminal narrowing. All participants in Study-3 selected North European, Eastern European, or ‘other Caucasian’ as the description of both their mother and father on the enrollment questionnaire. The demographic and risk factor characteristics of the participants in the 3 studies are presented in Table 1.

**Table 1:** Risk factors for MI in three case–control studies

	Study 1		Study 2		Study 3	
	Cases (n = 762)	Controls (n = 857)	Cases (n = 579)	Controls (n = 1159)	Cases (n = 475)	Controls (n = 619)
Male, %	60	41	81	42	61	62
Age at enrollment, median (range)	62 (29–87)	65 (24–100)	66(28–88)	58 (45–97)	60 (32–86)	58 (37–88)
Age at MI, median (range)	52 (27–82)	NA	57 (21–70)	NA	53 (29–77) †	NA
Smoking, %	66	45	68	40	73	54
Diabetes, %	20	0‡	25	0‡	38	10
Dyslipidemia§, %	84	53	84	61	95	56
Hypertension||, %	61	32	66	33	96	78
BMI (kg/m^2^), mean±SD	28±5	26±5	28±5	26±5	31±6	30±7

† Data available for 254 cases.

‡ Individuals with diabetes were excluded from control group.

§ Dyslipidemia was defined in Study 1 and Study 2 to be self-reported history of a physician diagnosis of dyslipidemia or the use of lipid lowering prescription medication(s) and defined in Study 3 to be the use of lipid lowering prescription medication(s), LDL cholesterol >129 mg/dL, triglycerides >149 mg/dL or HDL cholesterol <45 mg/dL .

|| Hypertension was defined in Study 1 and Study 2 to be a self–reported history of a physician diagnosis of hypertension or use of antihypertensive prescription medication(s) and defined in Study 3 to be the use of antihypertensive prescription medication(s), systolic blood pressure >160 mmHg, or diastolic blood pressure >90 mmHg.

NA; not applicable.

### SNP Selection

The 17,576 SNPs investigated in Study 1 are located in 10,152 genes. Of these 17,576 SNPs, 2767 were tested in at least one of 3 previously reported studies [8]–[10]. We previously reported that one of these SNPs (rs3798220 in LPA) was associated with cardiovascular disease [10]. Most of these SNPs (65%) could potentially affect gene function or expression because they cause an amino-acid change in a predicted open reading frame (missense SNPs), or they are located in exon acceptor or donor splice site, and could change the splicing pattern of predicted open reading frames. We also considered as potentially functional some SNPs that are located in regions that are known to be involved in transcriptional regulation (predicted transcription factor binding sites), or RNA stability (3′ or 5′ untranslated regions, or predicted microRNA binding sites).

### Allele Frequency and Genotype Determination

Initially, the allele frequency for each individual SNP was determined for all the cases and all the controls in pools of DNA. Pools were made by mixing equal volumes of standardized DNA from each individual member of the pool. Prior to pooling, DNA concentration for each sample was determined in triplicate using Picogreen fluorescent detection (Invitrogen). Measurements were repeated for samples which had high variation of fluorescence values (5% or greater coefficient of variation). DNA concentrations were determined from mean fluorescence values using a standard curve of salmon sperm DNA. DNA samples were then diluted to 6 ng/µL using automated liquid handling robotics (Beckman Coulter Fx, or Perkin Elmer Multiprobe II). The final concentration was confirmed using Picogreen fluorescent detection. Typically, several unique pools of DNA were made for cases and controls, made up of about 50 cases or controls. For each SNP, two real-time PCR reactions were performed, using 3 ng of pooled DNA in each reaction and allele-specific primers. The allele frequency in each pool was calculated from amplification curves for each allele. Genotyping of individual DNA samples was done by performing two real-time PCR reactions for each individual sample, using 0.3 ng DNA from each sample and allele specific primers.

### Ethics

Subjects of all three studies gave written informed consent and completed questionnaire approved by the Institutional Review Board of UCSF (Study 1 and Study 2) or the Cleveland Clinic Foundation (Study 3).

### Statistical methods

We assessed association between MI status and allele frequencies by two-tailed χ^2^ tests, and between MI status and genotype by logistic regression using an additive inheritance model (Wald test). In Study 2 and Study 3, since we tested a single prespecified risk allele for each SNP, we present one-sided P values and 90% confidence intervals (for odds ratios greater than one, there is 95% confidence that the true risk estimate is greater than the lower bound of a 90% confidence interval). We used a P threshold value of 0.05 in all three studies, and adjusted for multiple testing by calculating the False discovery rate (FDR) in Study 3. FDR was calculated using the MULTTEST procedure (SAS statistical package Version 9.1); for SNPs that were in the same gene, only the SNP with the higher (less significant) P value was included in the calculation.

## Results

We measured the allele frequencies of 17,576 putative functional SNPs in Study 1 cases and controls using pooled DNA samples and identified 1,949 SNPs that were associated with MI (P<0.05) and had minor allele frequency estimates that were greater than 1%. For these 1,949 SNPs, we determined allele frequencies in Study 2 cases and controls using pooled DNA samples and verified that the risk allele identified in Study 1 was also associated with risk of MI in Study 2. For those SNPs that were associated with MI and had the same risk alleles in both pooling studies, we then confirmed the association of the SNP with MI in Study 1 and Study 2 by genotyping individual DNA samples. We found that the risk alleles of 24 SNPs in 23 genes were associated with MI in both studies using an additive inheritance model (Table 2) and a P value threshold of 0.05. Next we tested the hypotheses that the risk alleles of these 24 SNPs would be associated with MI in Study 3. The power to detect association with MI for these 24 SNPs (based on the risk and allele frequency observed in Study 2) ranged from 41% (for rs3812475 in *TRMT12*) to 83% (for rs725660 in *LOC388553*). We found that the risk allele of 5 SNPs, in 4 genes (*ENO1, FXN* (2 SNPs), *HLA-DPB2,* and *LPA*) were associated with MI using an additive inheritance model after adjustment for age and sex (Table 3). The false discovery rate for these 5 SNPs was 0.23. The distribution of the genotypes for each of the SNPs did not deviate from what was expected under Hardy-Weinberg equilibrium (P>0.5). Further adjustment for traditional risk factors (dyslipidemia, hypertension, smoking status, and BMI), did not appreciably change the risk estimate for 4 of these SNPs (Table 3, *LPA, FXN* (2 SNPs), and *HLA-DPB2*). However, the risk for the *ENO1* SNP was not statistically significant after further adjustment for traditional risk factors (OR = 1.09, 90% CI 0.85–1.38, P = 0.28). Dyslipidemia could be confounding the association of the *ENO1* SNP with MI since this SNP trended toward association with dyslipidemia (P = 0.1).

**Table 2:** Twenty four SNPs associated with MI in Study 1 and Study 2

SNP	Gene Symbol	Risk Allele	Study 1				Study 2			
			Risk Allele Freq.	P value	OR	95%CI	Risk Allele Freq.	P value*	OR	90%CI
rs11568658	*ABCC4*	G	0.97	0.005	1.98	1.24–3.16	0.97	0.01	1.81	1.19–2.77
rs16875009	*ADAMTS16*	A	0.13	0.005	1.33	1.09–1.62	0.13	0.005	1.29	1.10–1.51
rs25651	*ANPEP*	T	0.29	0.0001	1.36	1.17–1.58	0.31	0.02	1.17	1.03–1.33
rs439401	*APOE*	T	0.35	0.03	1.17	1.01–1.35	0.37	0.01	1.19	1.05–1.35
rs867852	*C1orf81*	T	0.78	0.03	1.22	1.02–1.45	0.78	0.04	1.17	1.01–1.37
rs28372907	*DHX33*	A	0.18	0.03	1.21	1.02–1.44	0.18	0.01	1.22	1.05–1.42
rs11553576	*EML3*	T	0.60	0.03	1.17	1.02–1.35	0.60	0.03	1.16	1.02–1.31
rs1325920	*ENO1*	A	0.80	0.02	1.24	1.03–1.48	0.80	0.007	1.26	1.08–1.48
rs31208	*FAM71B*	G	0.11	0.03	1.27	1.03–1.57	0.12	0.006	1.30	1.09–1.55
rs3793456	*FXN*	G	0.56	0.01	1.20	1.05–1.39	0.55	0.03	1.15	1.02–1.30
rs10890	*FXN*	T	0.43	0.004	1.23	1.07–1.42	0.43	0.03	1.15	1.02–1.30
rs35410698	*HLA-DPB2*	G	0.93	0.02	1.43	1.06–1.91	0.94	0.006	1.53	1.16–2.03
rs1136141	*HSPA8*	G	0.86	0.05	1.24	1.00–1.53	0.86	0.02	1.26	1.05–1.52
rs7928656	*KCTD14*	A	0.84	0.04	1.23	1.01–1.51	0.84	0.004	1.32	1.11–1.57
rs3740918	*KIRREL3*	G	0.69	0.003	1.26	1.08–1.46	0.70	0.01	1.20	1.05–1.37
rs725660	*LOC388553*	A	0.34	0.006	1.23	1.06–1.42	0.35	0.0009	1.26	1.12–1.43
rs3798220	*LPA*	C	0.02	0.04	1.59	1.03–2.48	0.02	0.008	1.72	1.19–2.49
rs11711953	*MAP4*	T	0.07	0.03	1.34	1.03–1.73	0.07	0.01	1.35	1.09–1.67
rs4907956	*OLFM3*	G	0.60	0.03	1.18	1.02–1.36	0.61	0.01	1.19	1.05–1.34
rs2290819	*PTPRM*	T	0.38	0.03	1.17	1.01–1.35	0.38	0.008	1.20	1.06–1.35
rs3204635	*STAC3*	A	0.25	0.02	1.20	1.03–1.40	0.26	0.03	1.16	1.02–1.33
rs1866389	*THBS4*	C	0.20	0.03	1.21	1.02–1.43	0.20	0.03	1.19	1.03–1.37
rs3812475	*TRMT12*	T	0.50	0.03	1.16	1.01–1.34	0.52	0.04	1.13	1.01–1.28
rs862708	*ZNF304*	C	0.02	0.003	1.88	1.24–2.83	0.03	0.03	1.45	1.05–2.00

* 1 sided P value

**Table 3:** Genotypic association of 5 SNPs in Study 3

				Age and Sex adjusted			Fully Adjusted		
SNP (gene symbol)	Genotype	Cases n (%)	Controls n (%)	OR	90% CI	P value	OR	90% CI	P value
rs1325920	AA	327 (71)	394 (65)	1.61	0.92–2.81	0.08	1.28	0.62–2.62	0.3
(*ENO1*)	GA	120 (26)	186 (31)	1.25	0.70–2.22	0.3	1.20	0.57–2.54	0.3
	GG	14 (3)	27 (4)	ref			ref		
	Additive			1.28	1.06–1.55	0.01	1.09	0.85–1.38	0.3
rs10890	TT	117 (25)	98 (16)	1.52	1.14–2.04	0.009	1.49	1.02–2.18	0.04
(*FXN*)	CT	201 (44)	325 (54)	0.79	0.62–0.99	0.9	0.85	0.63–1.16	0.8
	CC	143 (31)	183 (30)	ref			ref		
	Additive			1.18	1.02–1.37	0.03	1.18	0.98–1.42	0.07
rs3793456	GG	174 (38)	180 (30)	1.33	0.98–1.80	0.06	1.50	1.01–2.22	0.04
(*FXN*)	AG	205 (45)	319 (53)	0.88	0.66–1.18	0.8	1.00	0.69–1.45	0.5
	AA	78 (17)	107 (18)	ref			ref		
	Additive			1.21	1.04–1.40	0.02	1.26	1.05–1.53	0.02
rs35410698	GG	426 (92)	539 (89)	1.56	1.09–2.22	0.02	2.07	1.31–3.27	0.004
(*HLA-DPB2*)	GA	36 (8)	70 (11)	ref			ref		
	Additive			1.46	1.03–2.06	0.04	1.79	1.14–2.81	0.02
rs3798220	CT	41 (9)	12 (2)	4.63	2.67–8.03	<0.001	3.52	1.85–6.69	0.001
(*LPA*)	TT	416 (91)	573 (98)	ref			ref		
	Additive			4.63	2.67–8.03	<0.001	3.52	1.85–6.69	0.001

## Discussion

We conducted an analysis of 17,576 SNPs that could potentially affect gene function or expression in three case-control studies of MI and identified 5 SNPs in four genes (*ENO1, FXN* (2 SNPs), *HLA-DPB2,* and *LPA)* that were associated with MI. The false discovery rate for this group of 5 SNPs was 0.23, indicating that several of these SNPs are expected to be associated with MI.

The first SNP is located in *ENO1,* a gene that encodes α-enolase, a glycolytic enzyme that catalyzes the conversion of 2-phospho-D-glycerate to phosphoenolpyruvate. α-enolase is also known to be a plasminogen receptor on the surface of hematopoietic cells and endothelial cells [11]. Thus, α-enolase could contribute to fibrinolysis, hemostasis, and arterial thrombus formation–processes that are critical in the pathophysiology of MI. The SNP in *ENO1* (rs1325920) is located about 1 kb upstream of the gene and could be involved in transcriptional regulation.

Two of the SNPs are in the *FXN* gene. The *FXN* gene encodes Frataxin, a mitochondrial protein involved in maintaining cellular iron homeostasis [12]. Expanded GAA triplet repeats in intron 1 of *FXN* leads to silencing of the *FXN* gene and to accumulation of iron in the mitochondria, which makes mitochondria sensitive to oxidative stress [13]. These changes lead to Friedreich's ataxia, an autosomal recessive disease of the central nervous system that is frequently associated hypertrophic cardiomyopathy [12]. The two SNPs in *FXN* found to be associated with MI are located in the 3′ untranslated region of *FXN* (rs10890) and in a putative transcription factor binding site (rs3793456), thus one or both of these SNPs could have an effect on *FXN* gene expression. These two SNPs are in linkage disequilibrium (r^2^ = 0.57 in Study 1) and thus, are not independent of one another. Whether these SNPs are associated with increased sensitivity of mitochondria to oxidative stress or to other mild manifestations of Friedreich's Ataxia symptoms is not known.

The fourth SNP (rs3798220 in *LPA*) encodes a isoleucine to methionine substitution at amino acid 4399 of apolipoprotein(a). We have previously shown that this SNP is associated with coronary artery narrowing and with increased levels of plasma lipoprotein(a) in case-control studies [10]. This SNP was also associated with incident myocardial infarction in the Cardiovascular Health Study, a population–based prospective study of about 5000 individuals aged 65 or older [14]. The low minor allele frequency of this SNP in LPA (1% in the European American population of CHS [14]) suggests that this SNP accounts for only a small fraction of the total variability of plasma Lp(a) levels. Our previous data showed that Lp(a) levels were 5.9-fold higher in carriers of the 4399 methionine allele than in noncarriers [10]; and high levels of Lp(a) are associated with an increased risk for MI [15], [16]. Additionally, one can speculate that the association of the 4399 methionine allele with increased risk of disease could also be due to the isoleucine to methionine change in apolipoprotein(a) that may result in a more deleterious form of Lp(a).

Lastly, the fifth SNP is in *HLA-DPB2* (rs35410698) is also associated with MI in this study. *HLA-DPB2* is a pseudogene in the Human Leukocyte Antigen (HLA) region. The HLA region is highly polymorphic, gene rich region. Linkage disequilibrium in this region can extend across hundred kilobases and encompass HLA as well as non-HLA genes [17]. Therefore, additional genotyping of SNPs in this region would be needed in order to know which gene variant in this region could be associated with MI.

### Limitations

We analyzed case–control studies that were retrospectively collected and did not include fatal cases of MI. Therefore, SNPs specifically associated with fatal MI would not have been identified. There were some differences between the participants in these three studies, specifically, Study 3 controls were recruited from patients who underwent coronary catheterization, whereas Study 1 and Study 2 controls were recruited from a lipid clinic population and from community centers. Thus, SNPs that were associated with MI in Study 1 and Study 2 but not in Study 3 might be explained by the differences between these studies. For example, a SNP in *THBS4* (rs1866389) that was found to be associated with MI in Study 1 and Study 2 but not in Study 3, has been previously reported to be associated with premature MI [18]. However, the power to detect the association of *THBS4* with MI in Study 3 was limited (40% power), thus the lack of association in Study 3 may represent a false negative result. The false discovery rate for the 5 SNPs that were associated with MI in Study 3 was 0.23. Thus we expect that some of the SNPs we identified could be false positive associations (type 1 error); replication from additional studies is required to validate the observed associations. We have looked for support for these associations in the published data from the Welcome Trust Case-Control Consortium data [19], unfortunately, none of the 5 SNPs we report here was genotyped in that study. Finally, although the SNPs in this study could potentially affect gene function, additional linkage disequilibrium analysis would be needed in order to determine if other SNPs in these region could better account for the associations with MI we observed.

### Conclusion

We identified 5 SNPs in 4 genes that are likely associated with MI. These SNPs merit investigation in additional studies of MI.
